# Left-Sided Colon Cancer and Right-Sided Colon Cancer: Are They the Same Cancer or Two Different Entities?

**DOI:** 10.7759/cureus.37563

**Published:** 2023-04-14

**Authors:** Mehdi Bourakkadi Idrissi, Hicham El Bouhaddouti, Ouadii Mouaqit, Abdelmalek Ousadden, Khalid Ait Taleb, El Bachir Benjelloun

**Affiliations:** 1 Department of Visceral Surgery, Hassan II University Hospital, Fez, MAR

**Keywords:** five-year overall survival, five-year disease-free survival, retrospective comparative study, right-sided colon cancer, left-sided colon cancer

## Abstract

Background

Colon cancer is one of the most common cancers in the world and one of the main causes of cancer-related deaths. In Morocco, it occupies the first place among digestive cancers. Right-sided and left-sided colon cancers have different embryological, epidemiological, pathological, genetic, and clinical characteristics. This distinction leads to differences in the evolution and prognosis of the disease. This study aimed to identify epidemiological factors and clinical and pathological characteristics that can influence perioperative and prognostic outcomes in patients with right-sided colon cancer compared to those with left-sided colon cancer.

Methodology

We conducted a retrospective cohort study over a period of nine years from January 2012 until December 2020. We included 277 patients divided into two groups, namely, right colon cancer (group 1) (n = 99) and left colon cancer (group 2) (n = 178).

Results

The average age of our series was 57.4 years, with extremes ranging from 19 to 89 years old (SD = ±13.6451 years). The average age in the right colon group was 55.97 (SD = ±13.341 years). The average age in the left colon group was 58.18 (SD = ±13.69 years). The male gender had a predominance, with a sex ratio of 1.3 for both groups. Among the patients in group 2, 65% showed lymph node involvement on the CT scan, whereas only 34% of patients in group 1 displayed the same condition. The recurrence rate in the right-sided colon cancer group was 22.2% compared to 24.9% in the left-sided group. The five-year overall survival was estimated for the right-sided and left-sided colon cancer groups at 87% and 96.5%, respectively. In patients with stage III and IV cancer, overall survival was better for those who underwent surgery for left-sided colon cancer compared to those who underwent surgery for right-sided colon cancer (p = 0.029). In the case of vascular emboli or involvement of the perineural sheath, there was no significant difference in overall survival (p = 0.446 and p = 0.655, respectively). The three-month survival without recurrence was almost identical in both groups (31% for right-sided colon cancers and 30.9% for left-sided colon cancers). Age over 61 years was a predictive factor of poor prognosis in recurrence-free survival (hazard ratio = 3.245; p = 0.023).

Conclusions

We identified factors that can influence perioperative outcomes and prognosis in patients with right-sided colon cancer compared to those with left-sided colon cancer. Our findings suggest that age and lymph node involvement along with other factors play a role in the overall survival and recurrence outcomes of these patients. Further research is necessary to explore these differences and develop personalized treatment plans for patients with colon cancer.

## Introduction

Colon cancer is one of the most common cancers in the world. It is one of the main causes of cancer-related deaths. It includes all malignant tumors that develop in the portion of the large intestine located between the ileocecal valve and the rectosigmoid junction. The frequency of colon cancer is high in most high-income countries where it constitutes a major public health problem, with an incidence of 1.4 million new cases per year worldwide, which represents approximately 15% of all cancers [1,2].

In France, it ranks first in cancer pathologies and represents nearly 15% of all cancers [1]. In Morocco, according to a study conducted at the National Institute of Oncology (INO) in Rabat, colon cancer occupies the first place among digestive cancers in Morocco (40.3%) [3], and according to the cancer registry in Fès [4,5], it represents 8.30% of all cancers in general and 38.82% of digestive cancers. Despite tangible progress made in recent decades in the field of diagnostic explorations, it remains underestimated and its mortality rate remains high [6]. Its incidence varies from one country to another due to differences in the lifestyle of populations, the type of diet, and hereditary predisposition. However, a common observation is that a younger population is starting to be affected by this type of cancer [7].

The classification of colon cancers is based on the location of the tumor, which results in the following two types: right-sided (proximal) colon cancers and left-sided (distal) colon cancers. Right-sided and left-sided colon cancers have different embryological, epidemiological, pathological, genetic, and clinical characteristics. This distinction leads to differences in the evolution and prognosis of the disease [8]. Consequently, it seems reasonable that colon cancers should be evaluated in two distinct categories [8,9].

Few studies have focused on analyzing this distinction, and, to date, colon cancers continue to be managed as a single entity. This study aimed to identify epidemiological factors, clinical and pathological characteristics, and therapeutic modalities that can influence perioperative and prognostic outcomes in patients with right-sided colon cancer compared to those with left-sided colon cancer and to compare them to the data from the literature.

This article was previously presented as a meeting abstract at the Berlin Digital Pathology Conference in April 2023.

## Materials and methods

This retrospective cohort study was conducted at the University Hospital Hassan II of Fez in the visceral surgery departments A and B over nine years from January 2012 to December 2020. The initial study population included all patients diagnosed with colon cancer, totaling 350. Inclusion criteria were symptomatic or complicated colon cancer patients admitted by appointment or emergency. Exclusion criteria consisted of tumors with mid-transverse and appendicular locations, patients with synchronous tumors or double locations, and patients eligible for immediate palliative treatment. In addition, we excluded patients with missing or incomplete files. After applying the inclusion and exclusion criteria, 277 patients were included and divided into two groups.

Data collection

The consultation records of patients were collected and organized through various sources, including hospitalization records for those experiencing obstructive symptoms in the colon, medical records, operative reports registry, and histopathological examinations registry. Additionally, the Hosix computerized data collection system of Hassan II University Hospital was utilized by using the IP addresses of selected patients and the surgical intervention code obtained from the General Register of Professional Acts (NGAP).

Statistical analysis

Patient data were coded and entered in a Microsoft Excel file. After validation, statistical analysis was performed using the statistical analysis software Epi Info in three steps. The first step involved a descriptive analysis of the collected data. The results were presented in the form of percentages and means ± standard deviation (SD). The second step involved univariate analysis, allowing for the comparison of means and percentages using statistical tests such as the Student’s t-test and chi-square test. The third step involved multivariate analysis using the stepwise logistic regression method. The results were reported in the form of commented graphs and tables. A p-value less than 0.05 was considered to be statistically significant.

Important definitions

The start of follow-up was defined as the date of the first histopathological result confirming the positive diagnosis of colon cancer. The date of recurrence was defined as the day when relapse was diagnosed either radiologically by identifying a local relapse, remotely by the appearance of metastasis, or by the result of a second biopsy. The date of loss to follow-up was considered as the date of the last consultation or, failing that, the date of the last imaging performed as part of the follow-up. Overall survival was defined as the time between the diagnosis of colon cancer and the occurrence of death regardless of the cause. Survival without recurrence was calculated from the date of the end of treatment (whether it was surgery alone or with adjuvant treatment) to the date of local recurrence or disease progression. Survival was studied as overall five-year survival and five-year disease-free survival. It was calculated using the Kaplan-Meier method. Survival curves were compared using the log-rank test. The significance level was set at 5%.

## Results

Sample size

A total of 350 patients of all ages were treated in the two visceral surgery departments during the study period. We included 277 patients in our study, as some files were missing or incomplete, and some cases were excluded due to criteria such as transverse colon cancer and concomitant cancers.

**Figure 1:**
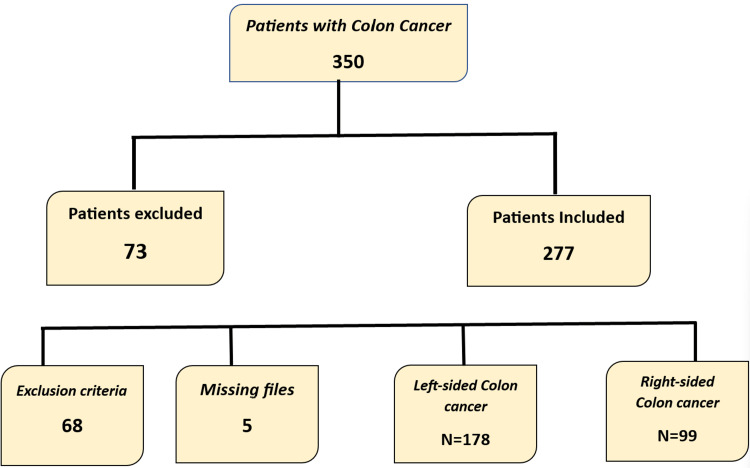
Patients’ selection process.

We divided our study sample into the following two groups: group 1 (n = 99) representing right-sided colon tumors, and group 2 (n = 178) representing left-sided colon tumors (Figure 1).

**Figure 2:**
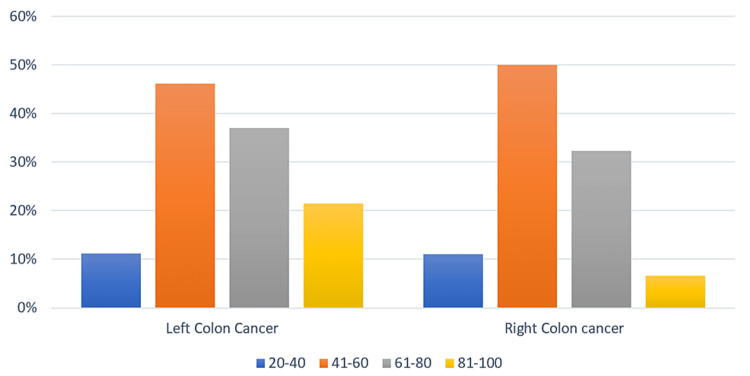
Distribution of patients by age category.

The average age of our series was 57.4 years, with extremes ranging from 19 to 89 years (SD = ±13.6451 years). The average age in the right colon group was 55.97 years (SD = ±13.341 years). The average age in the left colon group was 58.18 years (SD = ±13.69) (p = 0.614) (Figure 2).

The male gender had a predominance with a sex ratio (M:F) of 1.3 (4:3 four males for every three females) for both groups in our study. There were very few patients in our series who had risk factors or precancerous conditions. Of the total patients, five had a history of familial adenomatous polyposis, with two belonging to the right-sided colon cancer group, and the remaining three belonging to the left-sided colon cancer group. Additionally, four patients in group 2 had a history of ulcerative colitis, while one patient in group 1 was undergoing treatment for Crohn’s disease.

In total, 10 of our patients had at least one first-degree parent diagnosed with colorectal cancer. Seven patients belonged to group 2 and three patients belonged to group 1 (p = 0.7). A history of hereditary nonpolyposis colorectal cancer syndrome was found in three patients, with two belonging to group 2 and one to group 1 (p = 0.93).

**Figure 3:**
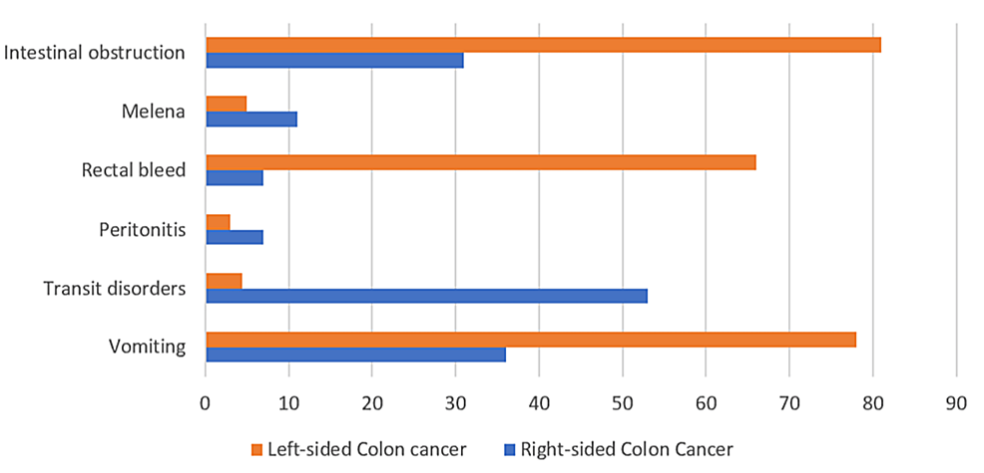
Signs and symptoms reported according to the location of the tumor.

For left-sided colon cancers, symptoms of bowel obstruction were the most common, followed by rectal bleeding. For right-sided colon tumors, bowel disorders (constipation, diarrhea, or obstruction) were the main symptoms (Figure 3).

Carcinoembryonic antigen (CEA) and carbohydrate antigen 19-9 (CA 19-9) were measured in 88% of patients. CEA was elevated in 32% of patients in group 2 and 29% of patients in group 1. CA 19-9 was elevated in 13% of patients in group 2 compared to 26% in group 1 (p = 0.016).

**Figure 4:**
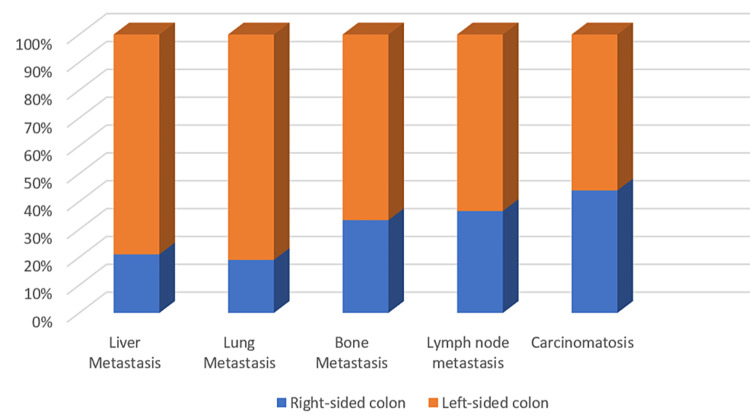
The distribution of metastatic cases according to the location of the primary tumor.

Among the patients in group 2, 65% showed lymph node involvement on the CT scan, whereas only 34% of patients in group 1 displayed the same condition. The hematogenous spread of cancer in our series is summarized in Figure 4.

The demographic data, medical history, and radiological findings are presented in Table 1.

**Table 1:** Summary of demographics, background, and CT findings.

	Right-sided colon cancer (n = 99)	Left-sided colon cancer (n = 178)	P-Value
Age	55.97 ± 13.341	58.18 ± 13.69	0.614
Sex ratio (M: F)	1.3 (4:3)	1.3 (4:3)	0.977
Comorbidities	20%	20.7%	0.538
Anemia	63%	46%	0.005
Risk factors			
Familial adenomatous polyposis	2	3	N/S
Chronic inflammatory bowel diseases	1	4	N/S
Family history of cancer			
First-degree parent diagnosed with colorectal cancer	3	7	0.7
Hereditary nonpolyposis colorectal cancer syndrome	1	2	0.93
High carcinoembryonic antigen	29.8%	32%	0.373
High carbohydrate antigen 19-9	26%	32.9%	0.016
Lymph node status +	34%	65%	0.551
Tumor stage			
T2	7.14%	7.9%	0.066
T3	65%	76%	0.071
T4	27%	15%	0.052

Postoperative outcomes were simple and without complications in 80.22% of patients who underwent surgery for left colon cancer and in 80.8% of patients who underwent surgery for right colon cancer. However, we noted the occurrence of postoperative complications in the remaining 20% of the cases which we divided into two categories, namely, major complications and minor complications. In major complications, we mainly included anastomotic leaks and postoperative peritonitis regardless of the etiology. Only 15 patients required a surgical re-intervention, with four patients belonging to group 2 and 11 belonging to group 1 (p = 0.03).

We considered recurrence to be a re-appearance of the disease at the local level (such as at an anastomosis site or on a colonic segment), as well as the appearance of a secondary distant or peritoneal location (carcinosis) after curative treatment. The recurrence rate in the right-sided colon cancer group was 22.2% compared to 24.9% in the left-sided group (p = 0.427).

Overall survival

The median of overall survival was estimated at 110 months with a 95% confidence interval of 35-18 for the right-sided colon cancer group. For the left-sided colon cancer group, it was estimated at 100 months with a 95% confidence interval of 93-107.

**Figure 5:**
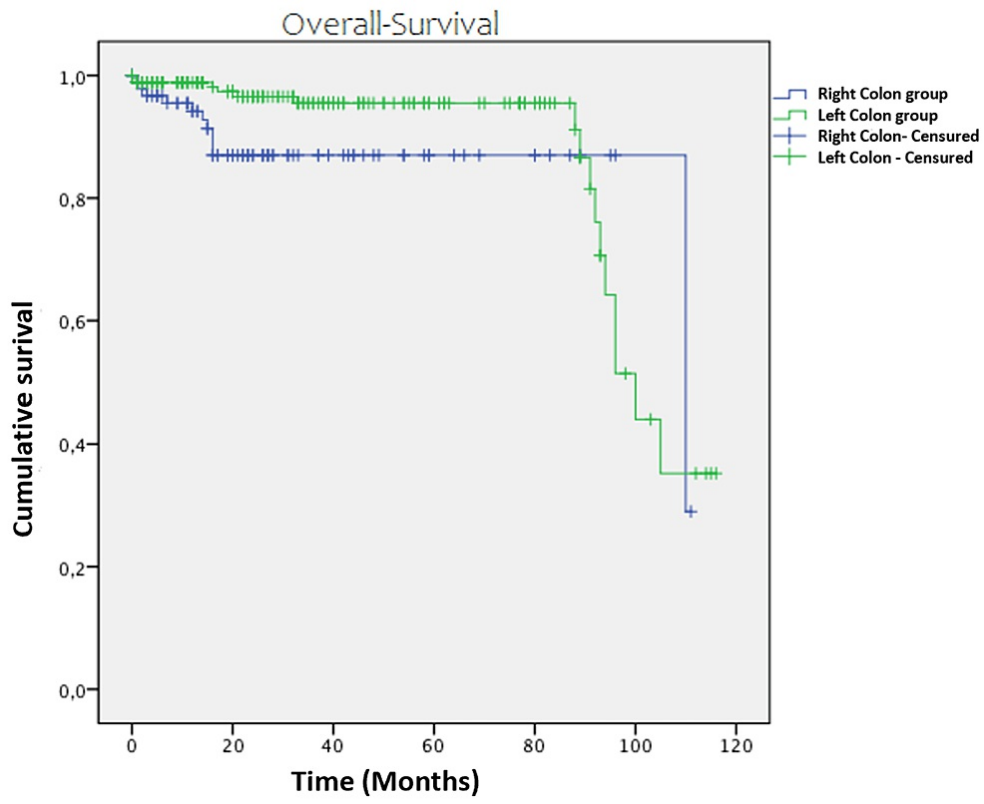
Kaplan-Meier curve representing overall survival over time in months for patients undergoing surgery for right-sided and left-sided colon cancer.

The five-year overall survival was estimated for the right-sided and left-sided colon cancer groups at 87% and 96.5%, respectively (p = 0.173) (Figure 5).

Postoperative outcomes and disease progression data are collated in Table 2.

**Table 2:** Summary of postoperative outcomes and evolution.

	Right-sided colon cancer (n = 99)	Left-sided colon cancer (n = 178)	P-value
Stay in the intensive care unit	1.47 (±2,607)	1.98 (±2.0959)	0.277
Length of hospital stay	10.56	8.5	0.872
Major complications	14%	7.86%	0.075
Degree of differentiation			
Well-differentiated	46.4%	57.3%	0.161
Moderately differentiated	31.3%	30.3%	0.366
Poorly differentiated	6%	0%	0.001
Presence of vascular emboli	22.2%	3.37%	0.001
Involvement of the perineural sheath	6%	0.56%	0.005
Recurrence	22.2%	16%	0.427
Mortality	12%	8.95	0.738
Overall survival	87%	96.5%	0.173
Disease-free survival	31%	30.9%	0.322

**Figure 6:**
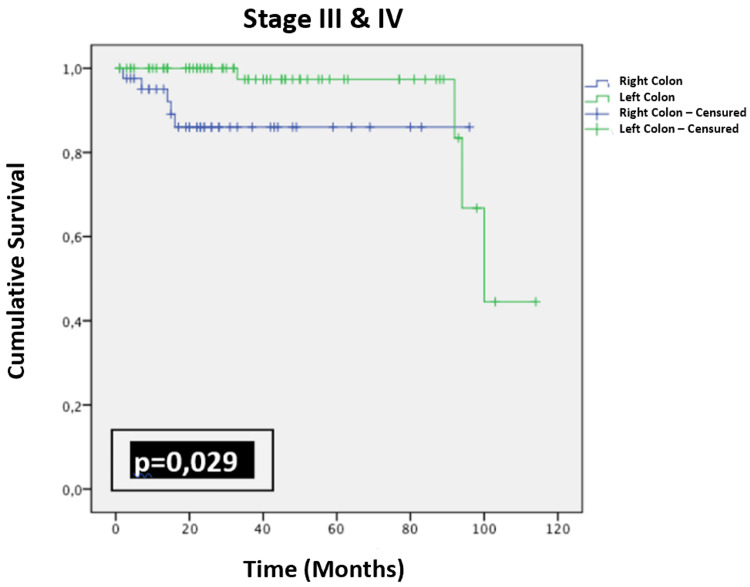
Kaplan-Meier curve representing overall survival over time in months for patients with stage III and IV colon cancer.

Our aim was to investigate the various factors that may impact overall survival. The comparison of the two overall survival curves across different age and sex groups did not show a statistically significant difference. There was no statistically significant difference in overall survival among patients who underwent surgery for stage I and II in both cancer groups (p = 0.263). However, among patients with stage III and IV cancer, overall survival was better for those who underwent surgery for left-sided colon cancer compared to those who underwent surgery for right-sided colon cancer (p = 0.029) (Figure 6).

**Figure 7:**
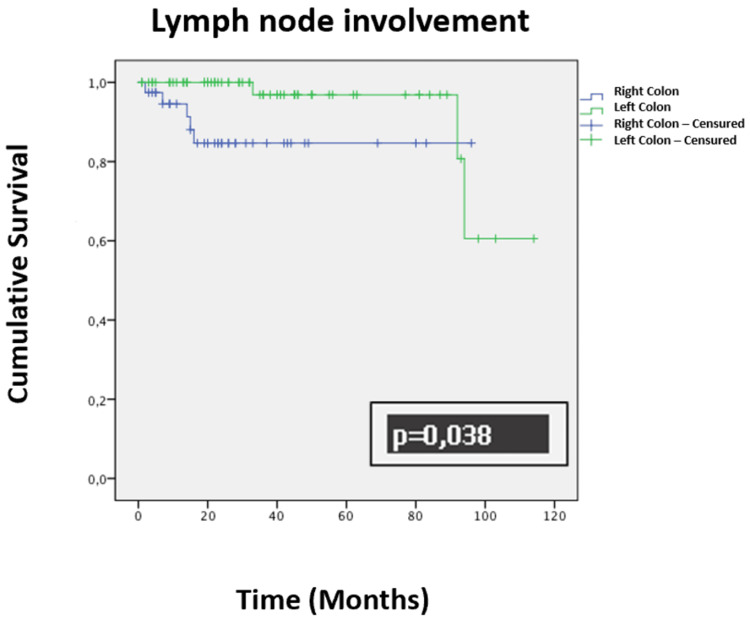
Kaplan-Meier curve representing overall survival over time in months for patients with lymph node involvement.

There was no difference in overall survival among patients who underwent surgery for right and left colon cancer, despite the occurrence of complications (p = 0.856). If lymph nodes were affected, the overall survival was better for patients who underwent surgery for left-sided colon cancer compared to those who underwent surgery for right-sided colon cancer (p = 0.038) (Figure 7).

In the case of vascular emboli or involvement of the perineural sheath, there was no significant difference in overall survival (p = 0.446 and p = 0.655, respectively).

The comparison of the two overall survival curves in different groups did not show a statistically significant difference according to the degree of differentiation.

Survival without recurrence

The median survival without events was estimated at 52 months with a 95% confidence interval of 34-18 for the right-sided colon cancer group and 49 months with a 95% confidence interval of 40-89 for the left-sided colon cancer group.

**Figure 8:**
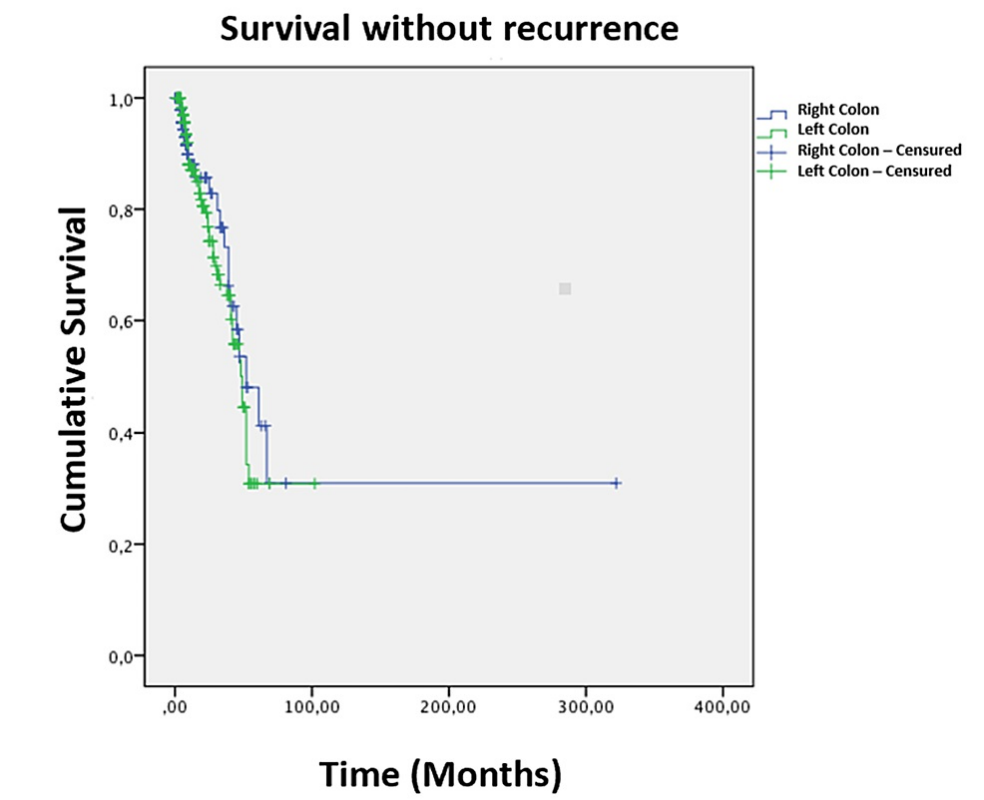
Kaplan-Meier curve representing overall survival over time in months for patients undergoing surgery for right-sided and left-sided colon cancer.

The three-month survival without recurrence was almost identical for both groups (31% for the right-sided colon cancer group and 30.9% for the left-sided colon cancer group) (p = 0.322) (Figure 8).

Survival without recurrence was better in patients with stage I or II left-sided colon tumor compared to those in the right-sided colon cancer group (p = 0.026). We were unable to find a difference in survival without recurrence between patients in the two groups with tumors classified as stage III or IV (p = 0.139).

Disease-free survival was better for both groups in the absence of major complications, although it was not statistically significant (p = 0.450).

There was no statistically significant difference in recurrence-free survival in cases of lymph node involvement (p = 0.181). Similarly, the presence of vascular emboli did not have a significant impact on recurrence-free survival, with no difference observed (p = 0.446 and p = 0.486, respectively).

## Discussion

Recent studies have delved into distinguishing left and right colon cancers as two separate entities in an attempt to uncover the differences in their pathophysiology, genetics, and, ultimately, prognosis [10-13]. These dissimilarities in clinical characteristics, anatomical features, and embryological origins can inform new and different approaches for patients based on the location of the colon tumor. To our knowledge, there have been limited studies of this nature conducted in the African continent. Therefore, it is crucial for us to undertake such research to gain additional insights into this topic.

The digestive system arises from the primitive gut during the delimitation stage of embryonic development, which occurs in the fourth week. This primitive gut comprises three parts. The foregut generates the esophagus, stomach, proximal duodenum, liver, biliary tract, and pancreas; the midgut creates the distal duodenum, jejunum, ileum, cecum, ascending colon, and two-thirds of the transverse colon; and the hindgut gives rise to the distal third of the transverse colon, descending colon, sigmoid colon, and rectum. This difference in origin translates into a dual vascular supply which can serve as the basis for all current hypotheses aimed at dividing colonic cancers into two distinct units [12,14].

Recent research has also demonstrated specific differences in the molecular biologic pattern between right-sided and left-sided colon cancers. A study using DNA microarray technology showed that there are more than 1,000 genes expressed differently between the right colon and the left colon [15]. Right-sided colon cancer is associated with three types of genes, namely, *MMR*, *K-RAS*, and *BRAF*, while left-sided colon cancer is associated with the expression of *CIN *and *p53* genes, NRAS, miRNA-146a, miRNA-147b, and miRNA-1288. Right-sided colon cancer is also associated with a high rate of microsatellite instability [16,17].

Because of these apparent differences that have been demonstrated, the idea that right-sided colon cancer and left-sided colon cancer are different tumors grows stronger but still fails to make its way into scientific guidelines, screening, and routine patient care.

Earlier studies found that patients with right-sided colon cancer were more likely to be older and female [10,18]. Other authors could not confirm this and reported no difference in age and sex [19,20]. The mean age in our series for right-sided colon cancer was estimated to be 55.97 years, while that for left-sided colon cancer was 58.18 years. This age is still significantly younger than the study by Mik et al. [21], Moritani et al. [22], and Benedix et al. [11], and similar to the results reported in a study conducted at the Pierre and Marie Curie Center in Algiers [23]. We noticed this high frequency in young patients in other Moroccan registries as well [5]. This age disparity in the Maghreb region can mainly be attributed to environmental and dietary factors but can also reflect the natural distribution of the age pyramid in these countries.

Right and left colectomies for colon cancer differ in multiple aspects. Their peri and postoperative outcomes have not been the subject of many investigations. In our series, we were able to report a high rate of major complications in right-sided colon cancer, although it was not statistically significant (14.14% for right-sided colon vs. 7.86% for left-sided colon, p=0.75). Our results are consistent with those of Benamr et al. [24], who reported a morbidity rate of 15.7%, dominated by postoperative peritonitis and fistulas.

In terms of anastomotic leakage, we noted a statistically significant difference between the two groups (p = 0.05), with more leakage after right colectomy (6.06 vs. 2.8%). These results are consistent with those reported by Mik et al. [21]. The rate of complications requiring surgical reoperation was higher in right-sided colon cancer (G1: 11.11% vs. G2: 2.24%, p = 0.003). These findings go against the common belief that right colectomies are easier and safer to perform compared to left colectomies with lower morbidity.

Despite all therapeutic advancements, tumor recurrence remains a major problem for all types of cancer, especially colorectal cancer. In other studies, the rate of tumor recurrence ranged between 4% and 16% [8,11,13,21,25].

The overall recurrence rate in our series was 22.2% for right colon cancer versus 16% for left colon cancer (p = 0.427). This is consistent with the literature, as reported by Staib et al. [26], with a recurrence rate of around 26.6%. However, the rate of local recurrence was estimated in the same analysis to be 3.4%, which is similar to our series with a local recurrence rate of 4% for the right colon compared to 1.68% for the left colon.

Survival analysis

The overall five-year survival rate varies among studies ranging from 16.5% to 77%. According to Benedix et al. [11], the five-year survival rate was higher for patients with left-sided colon cancer (71% for the left colon vs. 67% for the right colon; p = 0.01). These results are consistent with the recent findings of Meguid et al. [27], who analyzed 77,987 patients with colon cancer and found that right-sided colon cancer had a worse prognosis than left-sided colon cancer. However, studies with a smaller sample such as the one conducted by Gervaz et al. (n = 87) [8] have demonstrated a relatively better survival rate for right-sided colon cancer.

In our series, the average survival for right-sided colon cancer was 110 months compared to 100 months for left-sided colon cancer, with a better overall survival rate for left-sided cancer (87% for right-sided colon cancer vs. 96.5% for left-sided colon cancer, p = 0.07). Our results are comparable to those of several studies. It would seem more logical that the prognosis for right-sided colon cancer is more pejorative, considering the fact that the majority of right-sided colon cancers are poorly differentiated tumors, often locally advanced, and with a significant lymph node involvement rate [9,28].

The analysis of recurrence-free survival showed a minimal difference between the two groups [11]. Our series confirmed the same results. The median recurrence-free survival was almost the same (52 months for right-sided colon cancer and 49 months for left-sided colon cancer), with an identical recurrence rate at three months calculated at 31% for right-sided colon cancer versus 30.9% for left-sided colon cancer (p = 0.427). One possible explanation can be the two distinct pathways these cancers take to metastasize.

It is important to highlight that one of the main limitations of our study is the lack of complete follow-up data for more than 35% of the patients included in our sample. This factor could negatively impact our results regarding the outcome.

Prognostic factors and survival

Studying prognostic factors allows clinicians to adapt treatments to specific patients and better surveillance protocols. The stage of tumor progression at the time of diagnosis remains the main prognostic factor [26]. Nonetheless, it is crucial to define all factors conditioning survival within the same stage. These factors in colon cancers are clinical, biological, anatomopathological, and genetic.

Among the clinical prognostic factors frequently studied in the literature are age, sex, and the presence of complications. Age is a highly debated prognostic factor, with six out of 15 studies evaluating this factor, concluding that the onset of colon cancer in an elderly patient is a poor prognostic factor [10]. According to Helvaci et al. [25], the five-year survival rate for patients under the age of 75 was 58% and 32% for patients over the age of 75, with a significant difference. According to Arfa et al. [29], there is no significant difference in survival among different age groups. Meguid et al. [27] found that the risk of mortality increases by 3.6% each year (hazard ratio (HR) = 1.150). In our series, age did not have a significant impact on overall survival. The comparison of the two overall survival curves in different age groups showed no statistically significant difference according to age. However, a multivariate analysis by Cox regression was performed on our sample and showed that age over 61 years was a predictive factor of poor prognosis in recurrence-free survival (HR = 3.245; p = 0.023).

The prognosis of colon cancers operated at the stage of complications is more negative than cancers operated electively. The five-year survival rate is four to seven times lower than that of same-stage cancers operated under normal conditions [11]. In our study, the occurrence of major postoperative complications did not significantly impact overall survival (p = 0.856), nor did it impact survival without recurrence (p = 0.450).

The location of the tumor, whether on the left or right side, has an impact on prognosis and survival. The risk of recurrence is higher in patients with right-sided colon cancer. Right-sided colon cancer is associated with a poorer prognosis and lower survival rate compared to left-sided colon cancer. According to Meguid et al. [27], patients treated for right-sided colon cancer have a 4.2% higher risk of mortality compared to those treated for left-sided colon cancer (HR = 1.042, p = 0.001). However, there are some studies, such as that of Arfa et al. [29], which concluded the opposite. In our series, overall survival was better for left-sided colon cancers, although the finding was not significant.

The tumor stage affects the risk of death, with higher stages linked to higher mortality rates. In the study by Meguid et al. [27], the risk of mortality was 31% higher between stages I and II. It increased by 120% between stage II and stage III and reached 900% between stage III and stage IV. In the study by Arfa et al. [29], stage IV had the lowest two-year survival rate (22.7%), while stages I and II had a better two-year survival rate (85.3%). Survival was reduced in patients with a right-sided colon tumor classified as stage III and IV in the study by Hu et al. [30] (66.1% vs. 75.4%, p = 0.010). These results are consistent with our study. In our series, overall survival for tumors classified as stage III and IV was better for left-sided colon cancers (p = 0.029), as was the case for recurrence-free survival, which was better in patients with left-sided colon cancer classified as stage I or II (p = 0.026). Therefore, we can conclude that tumor stage was a predictive factor in the prognosis of colon cancer in our study.

The most common poor prognostic factor in all series is the low degree of differentiation compared to other histological types. It is found in about 34% of patients [28,29]. In our series, we had a low rate of poorly differentiated adenocarcinoma (2.40%). In our multivariate analysis, the impact of differentiation grade on survival was not statistically significant. Perhaps this is another limitation of our study. Because we included all types of colon tumors, not only adenocarcinomas (even though they accounted for 83% of our patients), we had a very small rate of poorly differentiated carcinomas (6% all in the right-sided colon cancer group).

Further prognostic analysis included perineural invasion and vascular emboli. Perineural invasion is considered an independent risk factor for locoregional recurrence in colon cancer [25]. Some studies did not report a difference between right-sided colon cancer and left-sided colon cancer regarding the presence of perineural invasion, whereas others reported a higher rate in patients with right-sided colon cancer (p = 0.05) [27,29]. For our population, perineural invasions were more prevalent in the right-sided colon cancer group (p = 0.005). However, we could not establish a link between their presence and overall survival (p = 0.655) or recurrence-free survival (p = 0.486). Vascular emboli have been found to be more frequent in right-sided colon cancers in several studies [25,27,29]. Our series confirmed these results as they were present in right-sided cancers (p = 0.01), but they did not have an impact on overall survival or recurrence-free survival (p = 0.446).

Nodal involvement is also a prognostic factor, with five-year survival rates dropping from 70% in the absence of nodal involvement to 11% in the presence of distant nodal involvement [28]. In our univariate analysis, the presence of nodal involvement was not significant for survival (p = 0.181); however, in our multivariate analysis using Cox regression, nodal involvement was retained as a predictive factor for recurrence (HR = 4.536; p < 0.001).

In our study, we observed significant differences between left-sided colon cancer and right-sided colon cancer. Additionally, we identified some prognostic factors that support the existing evidence for dividing colon cancer into two distinct categories. However, because our study included a limited number of patients, even the slightly different results between right-sided colon cancer and left-sided colon cancer turned out to be statistically significant. This could be another limitation of our study. While this significance should be taken into consideration, clinical significance should, nonetheless, be carefully evaluated.

## Conclusions

Colon cancer is still treated as a single disease, despite numerous studies highlighting the differences between left-sided and right-sided colon cancer. Our research has confirmed variations in epidemiological factors, complications, and overall survival between the two locations. In particular, we demonstrated that right-sided colon cancer is associated with a poor prognosis and that age, tumoral stage, and nodal involvement are predictive factors of a bad prognosis.

The findings suggest that colon cancer should be separated into distinct diseases, given that the left and right colons arise from different origins, follow different disease courses, and exhibit distinct molecular patterns, resulting in dissimilar prognoses. Therefore, identifying and treating left and right colon cancer as separate diseases can lead to more targeted and effective treatments, resulting in better patient outcomes.
